# Carbapenem-resistant *Enterobacteriaceae* (CRE) and gram-negative bacterial infections in south-west Nigeria: a retrospective epidemiological surveillance study

**DOI:** 10.3934/publichealth.2020062

**Published:** 2020-10-16

**Authors:** Oluwafolajimi Adetoye Adesanya, Hilda Amauche Igwe

**Affiliations:** 1 Institute for Advanced Medical Research and Training (IAMRAT), College of Medicine, University of Ibadan, Ibadan, Nigeria; 2 Department of Clinical Microbiology and Parasitology, College of Medicine, University of Ibadan, Ibadan, Nigeria

**Keywords:** carbapenem-resistance, extended spectrum β-lactamase, surveillance, multi-drug resistance, epidemiology, *Enterobacteriaceae*

## Abstract

**Background:**

Carbapenem-resistant *Enterobacteriaceae* (CRE) are often responsible for severe, life-threatening infections and they represent a critical threat to the available antibiotic agents and to global health. An understanding of the epidemiology of these infections will be indispensable to the development of appropriate case management as well as infection prevention and control (IPC) measures in any healthcare setting.

**Objectives:**

The objective of this study was to investigate and describe the epidemiology of carbapenem-resistant *Enterobacteriaceae* (CRE) and other gram-negative bacteria in a tertiary hospital in south west Nigeria using routinely collected microbiological laboratory data.

**Methods:**

A retrospective collection of microbiological laboratory records from the January to June 2018 was performed. All culture and antimicrobial susceptibility test results of patients who required laboratory tests were collected. Other information collected include: patient demographics, clinical specimen types and the requesting hospital department. The data was analyzed using SPSS Windows version 24. Comparison between categorical variables was done using chi-square tests while independent sample t-test was used to determine significant mean differences between groups. A *p* < 0.05 was taken to be statistically significant.

**Results:**

The prevalence of carbapenem-resistance among *Enterobacteriaceae* and gram-negative bacteria isolates was 22% (n = 39/177). Of these, 35.9% (n = 14) were *Klebsiella pneumonia*, 30.8% (n = 12) were *Pseudomonas aeruginosa* and 15.4% (n = 6) were *Klebsiella oxytoca*. 87.2% (n = 34) of these were also multi-drug resistant, with a mean total resistance score of 3.92 (SD = ± 1.44). There were differences observed in proportion of carbapenem-resistance across clinical specialties and age groups; however, these differences were not statistically significant. Independent sample t-test revealed that carbapenem-resistant isolates exhibited more drug resistance than carbapenem-sensitive isolates (3.93 vs. 2.30; *p* < 0.001).

**Conclusion:**

Carbapenem resistance is an important threat to the current antibiotic armory. Active surveillance, particularly in the healthcare setting is required to identify high risk groups, inform better treatment options and infection prevention and control measures.

## 1. Introduction

Antimicrobial resistance in commonly isolated bacteria has since been recognized by the World Health Organization (WHO) as a significant threat to global health, already reaching alarming levels in most parts of the world [1]. Of all the agents in the antimicrobial arsenal available to humans, carbapenems such as: imipenem, doripenem and etrapenem, are regarded as antibiotics of last resort, often reserved for infections resistant to the other commonly-used antibiotics. However, reports of carbapenem-resistant infections, especially those caused by the *Enterobacteriaceae* and other gram-negative bacteria have emerged, raising valid concerns that we may have reached the end of the antibiotics pipeline. Carbapenem-resistant *Enterobacteriaceae* are extremely virulent organisms exhibiting resistance to carbapenems and most, if not all, of the other classes of antibiotics available today. As a result, they have been associated with a high mortality rate. In a 2017 study by Tamma et al. [2], they found that 32% of patients with CRE bloodstream infections died within 14 days. Several mechanisms have been used to explain the emergence of carbapenem resistance (Table 1), which may be classified into: intrinsic and acquired mechanisms. Intrinsic mechanisms include the production of chromosomal carbapenemases from class A serine carbapenemases [3], as seen in *Enterobacter cloacae*, *Serratia mercescens* and *Klebsiella sp*., development of efflux pumps, as seen in *Pseudomonas aeruginosa* and *Acinetobacter baumannii* or the reduction in the outer membrane permeability through loss of porins [3],[4].

The acquired mechanisms are often plasmid-mediated, spreading through horizontal gene transfer. Some of the carbapenemases implicated in the acquired mechanisms include: *Klebsiella pneumoniae* carbapenemase, which is one of the most frequently isolated carbapenemase globally, and has been identified as the cause of several nosocomial outbreaks [5]–[7]; and the New Delhi metallo-β-lactamase (NDM), first isolated in 2008, and now one of the most important carbapenemases today [8]. Over the last decade, the incidence of CRE infections has risen dramatically worldwide, however, while epidemiological data exists from developed settings in Europe [9], the USA [10],[11] and China [12], there remains a critical paucity of data from resource limited settings like those found in Africa. One of the most important strategies identified for the control of antimicrobial resistance is active disease surveillance, especially in the healthcare setting.

There is a need for expanded efforts towards the active surveillance of *Enterobacteriaceae* as well as other gram negative infections, in order to rapidly identify changing resistance patterns and emerging threats. One way in which such surveillance can be achieved is by collecting and analysing routinely collected medical health records, to highlight these changing trends and implement effective strategies towards clinical case management and infection prevention and control [13]. The aims of this study therefore were: to elucidate the epidemiology of CRE infections in a tertiary care facility in south-west Nigeria, from routinely-collected hospital microbiology data, and identify how factors such as age, gender and hospital location, influenced the chances of developing CRE infections in the study population. This would in turn; fill the identified knowledge gaps on the epidemiology of CRE infections in low resource settings, the data of which will also inform strategies for infection prevention and control.

**Table 1.** Mechanisms of emergence of carbapenem-resistance.

Type of mechanism	Examples
Through carbapenemases	Class A serine carbapenemases:
	- *Klebsiella pneumoniae* carbapenemases (KPC)
	- Guiana extended spectrum carbapenemases
	Class B metallo-β-lactamases (MBLs):
	- Verona integrin-encoded metallo-β-lactamase (VIM)
	- Sao Paulo metallo-β-lactamase (SPM)
	- Seoul imipenemases (SIM)
	- German imipenemases (GIM)
	- New Delhi metallo-β-lactamase (NDM)
	Class D serine carbapenemases:
	- OXAβ-lactamases (e.g. OXA-48 and its variants)
Through extended spectrum beta-lactamases (ESBL) or Amp-C beta-lactamase, with efflux pump development or cell surface porin loss	

## 2. Materials and method

### 2.1. Setting

A retrospective epidemiological surveillance study was conducted between January 1, 2018 and June 30, 2018 at the Department of Medical Microbiology and Parasitology of the University College Hospital (UCH), Ibadan. The University College Hospital (UCH) is a 1,000-bed tertiary care facility and university teaching hospital in Ibadan, south-west Nigeria, with approximately 150,000 new patients (in-patient and out-patients) every year [14]. It is the largest hospital in the region, offering care in all medical and surgical specialties, to the entire south-western region of Nigeria, and acts as a referral centre for most of the Nigerian population. The Department of Clinical Microbiology and Parasitology provides microbiological laboratory diagnostics procedures as well as molecular identification of microorganisms and serological testing services.

### 2.2. Study population

For this study, male and female patients who had routine laboratory microbiological investigations for bacterial infections (culture and sensitivity) during the study period were eligible. Exclusion criteria included: missing key data and isolates with mixed colonies suggestive of sample contamination. A case of CRE was defined a clinical culture with organisms of the *Enterobacteriaceae* family, exhibiting phenotypic resistance (determined using zone of inhibition diameter on Kirby-Bauer disk diffusion method, and according to the guidelines of the Clinical and Laboratory Standards Institute) to carbapenems (imipenem, meropenem or etrapenem). An initial literature review identified members of the *Enterobacteriaceae* family to be included in the study: *Klebsiella pneumoniae*, *Klebsiella oxytoca*, *Escherichia coli*, *Proteus mirabilis*, *Enterobacter sp*., *Morganella morganii* and *Providencia sp*. [15]. Other gram-negative infections included in the study include: *Pseudomonas aeruginosa*, *Pseudomonas luteola*, *Acinetobacter baumannii* and *Haemophilus influenza*.

### 2.3. Data collection

Records of the Clinical Microbiology and Parasitology department were reviewed to collect clinical and epidemiological data of patients meeting the inclusion criteria for the study. The data collected include: patient demographics, location in the hospital, samples collected, isolate types and results of antibiotic susceptibility testing. All data was extracted from the laboratory records using a pre-designed Microsoft Excel 2010 structured spreadsheet.

### 2.4. Bacterial species identification and antimicrobial susceptibility testing

Isolates were cultured using standard bacteriological techniques, and bacteria species were identified and confirmed using biochemical methods, such as: oxidase reaction, indole test, pigmentation, mucoid production and growth pattern on MacConkey agar on incubation, as well as with the use of the available miniaturized multi-test identification systems API (bio Merieux SA) and Microgen (Microbiology International, UK). Antimicrobial susceptibility testing was performed using the Kirby-Bauer disc diffusion method on Mueller-Hinton agar (Oxoid, England) and the interpretations were carried as described in the guidelines of the Clinical and Laboratory Standard Institute (CLSI) [16]. Antibiotics against which the isolates were tested include: ceftazidime (30 µg), ceftriaxone (30 µg), cefuroxime (30 µg), cefoxitin (30 µg), gentamicin (10 µg), amikacin, ampicillin (10 µg), erythromycin (15 µg), ciprofloxacin (5 µg), ofloxacin (5 µg), nitrofurantoin (300 µg), amoxicillin/clavulanic acid (Augmentin®) (30 µg), meropenem (10 µg), colistin (10 µg), ampicillin/sulbactam (10 µg/10 µg) and piperacillin/tazobactam (100 µg/10 µg). The plates were incubated at 37 ˚C for 24 hours and the diameters of the zones of complete inhibition were measured to the nearest millimeter and compared with the guidelines of the CLSI [16] to determine resistance and sensitivity states. *Escherichia coli* ATCC25922 and *Klebsiella pneumoniae* ATCC13883 were used as reference strains for the susceptibility testing.

### 2.5. Statistical data analysis

Prior to analysis, data was processed to ensure each row represented the susceptibility result for each patient, isolate or sample. Age was categorized as follows: 0–16, 17–24, 25–39, 40–54, 55–69, and ≥70 years old. Patient location (ward) was assigned to the corresponding clinical department. Total resistance scores were calculated for each isolate by adding the resistance scores obtained in each antibiotic category tested i.e. any isolate demonstrating resistance to at least one cephalosporin agent was assigned a resistance score of 1 in the cephalosporin resistance variable. Descriptive statistics were used summarize clinical and epidemiological characteristics of CRE infections. Continuous variables were presented as medians with their interquartile range while categorical variables were expressed as frequencies with their percentages. The *x*^2^ test of association was used to determine if there were statistically significant differences in the proportion of CRE infections between age groups, sex and clinical specialties or hospital sites. Independent sample t-test was performed to determine if mean differences between subjects of a categorical variable were statistically significant. The threshold for statistical significance was *p* < 0.05 Data analysis was done with the SPSS Windows version 24 (IBM, Chicago, IL, USA).

### 2.6. Ethical consideration

Ethical approval for the study was obtained from the Institutional Review Board of the University of Ibadan/University College Hospital (UI/UCH IRB). All data was annonymised prior to analysis and all data was treated with strict confidentiality. As the study did not directly involve human participants, the requirement for informed consent was waived.

## 3. Results

### 3.1. Patient characteristics, bacterial isolates and specimen culture

Between January 1, 2018 and June 30, 2018, a total of 800 samples were received by the clinical microbiology and parasitology department for antimicrobial culture and sensitivity testing, of which 235 (29.4%) yielded significant growth on bacterial culture. Of these, there were 177 (22.1%) microbiology reports made across all *Enterobacteriaceae* and gram-negative bacteria species included in analysis, the distribution of which is summarized in Table 2. These reports represented the data for 177 patients; of which 81 patients were females (45.8%) and 96 patients were males (54.2%), with a median age of 42 years (interquartile range, 29 to 64 years; Table 3). The four most frequently recorded specimens from which the *Enterobacteriaceae* and gram-negative organisms were cultured include: urine (n = 72; 40.7%), wound culture (n = 40; 28.3%), tracheal aspirate (n = 16; 9.0%) and sputum (n = 13; 7.3%) as summarized in Table 4. The clinical departments from which the highest number of patients from which *Enterobacteriaceae* and gram-negative organisms were cultured include: surgery (n = 54; 30.5%), medicine (n = 36; 20.3%), pediatrics (n = 25; 14.1%) and intensive care (n = 23; 13.0%).

**Table 2.** Distribution of organisms isolated during the study period.

Organism	Isolated; n (% = n/total isolate)	Carbapenem-susceptibility	
		Sensitive; n (% = n/total organism)	Resistant; n (% = n/total organism)
*Klebsiella pneumoniae*	59 (33.3)	45 (76.3)	14 (23.7)
*Escherichia coli*	36 (20.3)	34 (94.4)	2 (5.6)
*Pseudomonas aeruginosa*	31 (17.5)	19 (61.3)	12 (38.7)
*Klebsiella oxytoca*	24 (13.6)	18 (75.0)	6 (25.0)
*Proteus mirabilis*	12 (6.8)	11 (91.7)	1 (9.1)
*Enterobacter cloacae*.	3 (1.7)	3 (100)	0 (0.0)
*Acinetobacter baumannii*	3 (1.7)	1 (33.3)	2 (66.7)
*Proteus vulgaris*	3 (1.7)	3 (100)	0 (0.0)
*Morganella morganii*	2 (1.1)	2 (100)	0 (0.0)
*Pseudomonas luteola*	2 (1.1)	1 (50.0)	1 (50.0)
*Haemophilus influenza*	1 (0.6)	1 (100)	0 (0.0)
*Providencia sp*.	1 (0.6)	0 (0.0)	1 (100)
Total	177 (100)	138 (100)	39 (100)

**Table 3.** Demographic characteristics of patients included in the study.

Characteristics	Data		
	All *Enterobacteriaceae* and gram-negative isolates	Carbapenem-resistant *Enterobacteriaceae* and gram-negative isolates	*p* value
Sex	n (% = n/total isolate)	n (% = n/total resistant)	0.16
- Female	81 (45.8)	14 (35.9)	
- Male	96 (54.2)	25 (64.1)	
Age groups (years)	n (% = n/total isolate)	n (% = n/total resistant)	0.11
- 0–16	29 (16.4)	4 (10.3)	
- 17–24	12 (6.8)	1 (2.6)	
- 25–39	42 (23.7)	10 (25.6)	
- 40–54	36 (20.3)	14 (35.9)	
- 55–69	30 (16.9)	5 (12.8)	
- ≥ 70	28 (15.8)	5 (12.8)	
Total	177 (100)	39 (100)	

**Table 4.** Distribution of organisms across clinical specimens.

Specimen	Number of organisms		*p* value
	All *Enterobacteriaceae* and gram-negative bacteria; n (% = n/total isolates)	Carbapenem-resistant isolates; n (% = n/number of isolates in specimen)	
Urine	72 (40.7)	15 (20.8)	0.00
Wound culture	50 (28.3)	10 (20.0)	
Tracheal aspirate	16 (9.0)	7 (43.8)	
Sputum	13 (7.3)	4 (30.8)	
Catheter tip	7 (4.0)	3 (42.9)	
Pleural fluid	6 (3.4)	0 (0.0)	
Eye swab	4 (2.3)	0 (0.0)	
Pus	3 (1.7)	0 (0.0)	
Ear swab	2 (1.1)	0 (0.0)	
Tissue biopsy	2 (1.1)	0 (0.0)	
Rectal swab	1 (0.6)	0 (0.0)	
CSF	1 (0.6)	0 (0.0)	

### 3.2. Antimicrobial susceptibility testing

Of the 177 *Enterobacteriaceae* and gram-negative bacterial infections, 39 (22%) displayed phenotypic carbapenem resistance, representing 8 different bacterial species of the 12 species studied. Of these, *Klebsiella pneumoniae* (n = 14; 35.9%) and *Pseudomonas aeruginosa* (n = 12; 30.8%) displayed the highest levels of carbapenem resistance. The other strains detected include: *Klebsiella oxytoca* (n = 6/39; 15.4%), *Escherichia coli* (n = 2/39; 5.1%), *Proteus mirabilis* (n = 1/39; 2.6%), *Pseudomonas luteola* (n = 1/39; 2.6%) and *Providencia sp*. (n = 1/39; 2.6%). The results of *x*^2^ analysis showed that the difference in rates of carbapenem resistance across the bacterial strains was significant (*p* = 0.02), with *Klebsiella pneumoniae* and *Pseudomonas aeruginosa* strains showing significantly higher rates of carbapenem resistance, compared to other isolated strains as summarized in Table 2. A difference in the proportion of carbapenem resistance was noticed across clinical specialties, with surgery and the intensive care unit (ICU), displaying the highest rates of carbapenem resistance among the bacterial species studies at (n = 14/39; 35.9%) and (n = 9/39; 23.1%) respectively. However, *x*^2^ analysis did not suggest that this difference was statistically significant (*p* = 0.34), as displayed in Table 5. A similar difference in proportion of carbapenem resistance was observed across the patient age categories studied (Table 3), with the age range 40 – 54 years displaying the highest rate of carbapenem resistant infections (n = 14/39; 35.9%), while the age range 17–24 years displaying the least (n = 1/39; 0.26%). However upon *x*^2^ analysis, this difference was also found to be statistically insignificant (*p* = 0.11).

Furthermore, drug resistance analysis revealed that 34 (87.2%) of the carbapenem-resistant *Enterobacteriaceae* and gram-negative bacteria isolates were multi-drug resistant (defined as phenotypic resistance to at least one antibiotic agent in a minimum of three different antibiotic groups), compared to 62 (44.9%) of the carbapenem-sensitive isolates. Looking into the specific antibiotic agents tested, the carbapenem-resistant isolates exhibited the highest levels of phenotypic resistance to the cephalosporins (n = 33/39; 84.6%), followed by fluoroquinolones (n = 28/39; 71.8%) and aminoglycosides (n = 26/39; 66.7%), while 82.1% of isolates were susceptible to colistin (Table 6). The result of an independent sample t-test indicated that there was also a significant difference in total resistance scores between the carbapenem-sensitive and carbapenem-resistant *Enterobacteriaceae* and gram-negative bacteria isolates, t (175) = 6.72, *p* < 0.001, with the average total resistance score of the carbapenem-resistant isolates (*M* = 3.93, SD = 1.44) being significantly higher than that of the carbapenem-sensitive isolates (*M* = 2.30, SD = 1.30).

**Table 5.** Distribution of carbapenem-resistant *Enterobacteriaceae* and gram-negative isolates across clinical specialties.

Clinical specialties	Number of carbapenem-resistant *Enterobacteriaceae* (CRE) and gram-negative isolates; n (% = n/total resistant isolates)	*p* value
Surgery	14 (35.9)	0.34
Intensive care unit (ICU)	9 (23.1)	
Medicine	6 (15.4)	
Paediatrics	4 (10.3)	
Obstetrics & Gynaecology (O&G)	3 (7.7)	
Emergency department (ED)	2 (5.1)	
General out-patient department (GOPD)	1 (2.6)	

**Table 6.** Percentage of resistant CRE isolates by antibiotic class.

Antibiotics class	Number of carbapenem-resistant *Enterobacteriaceae* (CRE) and gram-negative isolates; n (% = n/total resistant isolates)
Cephalosporins	33 (84.6)
Aminoglycosides	26 (66.7)
Fluoroquinolones	28 (71.8)
Colistin	7 (17.9)
Nitrofurantoin	9 (23.1)
Penicillin	11 (28.2)

## 4. Discussion

Carbapenem-resistant *Enterobacteriaceae* and gram-negative infections have become a critical problem and a significant threat to global health, associated with a high morbidity and mortality rate [2],[11],[17]. Results from our epidemiological study placed the prevalence of carbapenem-resistance among *Enterobacteriaceae* and gram-negative bacteria isolates in the study location at 22%. This value lower than those reported in literature from other locations—Uganda (30.6% [18]), Tanzania (42.0% [19]), India (24.0% [20]), but higher than that reported from Tunisia (15.8% [21]). Other studies from different locations in Nigeria have also reported varying numbers with Shettima et al. [22] reporting a prevalence of 6.8% from Northeast Nigeria, while Ogbolu et al. [23] and Olalekan et al. [24] reported a prevalence of 36.8% and 27.4% respectively. The clinical samples from which the highest proportion of carbapenem-resistant isolates were cultured were urine (40.7%), wound culture (28.3%), tracheal aspirate (9.0%) and sputum (7.3%). These are very similar to the results obtained in a similar study in London by Freeman et al. [25], where urine cultures (44.5%), wound cultures (23.7%) and sputum cultures (14.5%) were the most frequently recorded clinical specimens containing carbapenem-resistant *Enterobacteriaceae* and gram-negative bacteria infections. Differences were also noticed in the proportion of carbapenem-resistant infections across clinical departments, with samples from patients on surgical wards as well as in the intensive care unit (ICU) displaying the highest proportions of carbapenem-resistance, a results which corresponds with that obtained in a similar study in South Africa by Thomas and Duse [26], where the ICU (5%) and surgical wards (23%) reported the highest CRE prevalence. This difference could be explained by the critical nature of the illnesses patients on these wards suffer and their requirement of prolonged hospitalization. This information could be used to identify locations within the hospital requiring increased infection prevention and control measures to reduce the dissemination of CRE and gram-negative infections.

Another variable within which there were differences in proportion of carbapenem-resistant isolates was age, with the age group 40–54 years displaying the highest proportion (35.9%) of CRE infections and the age group 17–24 years displaying the lowest proportion (0.26%). This finding is commensurate with those from other studies such as that from China by Zhang et al. [12], in which patients aged 50–64 years had the second highest rate of carbapenem-resistant infections (23.9%), while those aged 65–79 years had the highest rate (28.9%). However, a prospective, multicentre, observational study of hospitals in Ohio, Pennsylvania and Michigan, launched by the Consortium on resistance against carbapenems in *Klebsiella* and other *Enterobacteriaceae* (CRACKLE) found that the median age of patients enrolled in the study, with carbapenem-resistant *K. pneumoniae* infections was much higher at 70 years (interquartile range 58–81 years) [27]. A possible explanation for the higher predisposition of middle-aged and elderly patients to these infections could be the higher prevalence of co-morbidities such as: diabetes mellitus, hypertension, cardiovascular diseases, renal insufficiency and cancer, which result is frequent hospitalization, long hospital stay and use of multiple antibiotics use, all of which predisposes them to the most severe forms of these infections [28],[29]. As regards antibiotic susceptibility testing, 87.2% of the carbapenem-resistant isolates were also found to be multi-drug resistant (defined as non-susceptibility to at least one agent in three different antibiotic groups), as compared with 44.9% in the carbapenem-susceptible *Enterobacteriaceae* and gram-negative bacteria isolates included in the study.

Furthermore, of the groups of antibiotics tested, cephalosporins (resistance rate = 84.6%), fluoroquinolones (resistance rate = 71.8%) and aminoglycosides (resistance rate = 66.7%) had the least effect on the carbapenem-resistant isolates. These findings are in tandem with existing literature on the subject as reported by Zhang et al. [12], where all carbapenem-resistant *Enterobacteriaceae* were also resistant to all cephalosporins tested, and aminoglycosides possessed better activity compared with fluoroquinolones. The reason for this can be traced back to the mechanism of development of carbapenem-resistance, which is due to the development of extended spectrum-β-lactamases, and cephalosporins, being β-lactam antibiotics themselves would therefore be ineffective against CRE infections. Meanwhile, polymixins E (or colistin) had the highest effect, with 82.1% of the isolates being susceptible to the agent. Finally, through an independent sample t-test, we were able to confirm that carbapenem-resistance was indeed associated with a higher rate of antimicrobial resistance than carbapenem-susceptibility, in our study population.

With the rise in the rate of antimicrobial resistant infections in general, and carbapenem-resistant *Enterobacteriaceae* and gram-negative bacteria infections in particular, there have been calls for the urgent development of alternatives to antibiotics, for combating these infections. Some options being explored include the use of antimicrobial peptides (AMP) and ceragenins for treating these infections [30]. Some in vivo studies have shown the effectiveness of ceragenins and AMPs in eliminating bacterial isolates [31]. Another alternative being pushed is bacteriophage therapy and several in vitro and in vivo human and animal studies have shown that this approach could be effective. However there is need for further standardized clinical trials in humans to establish its safety and efficacy, as well as help navigate the many regulatory hurdles towards its entry into conventional human medicine [32].

## 5. Limitations

Results from the analysis presented in this study have provided insight into the epidemiology of carbapenem-resistant *Enterobacteriaceae* and other gram-negative bacteria infections in the healthcare setting being investigated. Furthermore, we have shown how routinely collected microbiological laboratory data can be used to develop a deeper understanding of infectious disease trends and emerging threats; however, this study is not without its limitations. First, we have not spoken about the molecular epidemiology of the mechanism of carbapenem-resistance in the study population. This would be crucial to further understand how the molecular basis of how these observed resistance patterns have emerged. Second, we also did not speak about the hospital-wide incidence of carbapenem-resistance as well as the clinical characteristics of the patients from which the isolates studied were obtained. This was because the required data could not be obtained due to the lack of a centralized electronic health information management system in the study location. Such data if available would provide insight into the clinical risk factors of carbapenem-resistance in the study population, which could inform health promotion efforts. Finally, due to the small sample size, we could not carry out an organism-level analysis, to determine the trends of carbapenem-resistance between bacterial species and investigate any differences or associations that may exist.

## 6. Conclusion

In conclusion, going forward, we recommend that a prospective, multi-centre epidemiological surveillance study be considered, involving multiple sites in different regions across the country, to better understand the patterns of carbapenem-resistance as well as other forms of antimicrobial resistance that may be developing across the country. We believe the findings from our study have the potential to influence far-reaching infection prevention and control measures towards curtailing the spread of carbapenem-resistance and safe-guarding our existing antibiotics armory.
